# Poisonous Plants of the Genus *Pimelea*: A Menace for the Australian Livestock Industry

**DOI:** 10.3390/toxins15060374

**Published:** 2023-06-02

**Authors:** Rashid Saleem, Ali Ahsan Bajwa, Shane Campbell, Mary T. Fletcher, Sundaravelpandian Kalaipandian, Steve W. Adkins

**Affiliations:** 1 School of Agriculture and Food Sciences, The University of Queensland, Gatton, QLD 4343, Australias.kalaipandian@uq.edu.au (S.K.); s.adkins@uq.edu.au (S.W.A.); 2 Weed Research Unit, New South Wales Department of Primary Industries, Wagga Wagga, NSW 2650, Australia; 3 Queensland Alliance for Agriculture and Food Innovation, The University of Queensland, Coopers Plains, QLD 4108, Australia; mary.fletcher@uq.edu.au; 4 Department of Bioengineering, Saveetha Institute of Medical and Technical Sciences (SIMATS), Saveetha School of Engineering, Chennai 602105, Tamil Nadu, India

**Keywords:** *Pimelea*, simplexin, animal toxicity, weed biology, weed management, herbicides

## Abstract

*Pimelea* is a genus of about 140 plant species, some of which are well-known for causing animal poisoning resulting in significant economic losses to the Australian livestock industry. The main poisonous species/subspecies include *Pimelea simplex* (subsp. *simplex* and subsp. *continua*), *P. trichostachya* and *P. elongata* (generally referred to as *Pimelea*). These plants contain a diterpenoid orthoester toxin, called simplexin. *Pimelea* poisoning is known to cause the death of cattle (*Bos taurus* and *B. indicus*) or weaken surviving animals. *Pimelea* species are well-adapted native plants, and their diaspores (single seeded fruits) possess variable degrees of dormancy. Hence, the diaspores do not generally germinate in the same recruitment event, which makes management difficult, necessitating the development of integrated management strategies based on infestation circumstances (e.g., size and density). For example, the integration of herbicides with physical control techniques, competitive pasture establishment and tactical grazing could be effective in some situations. However, such options have not been widely adopted at the field level to mitigate ongoing management challenges. This systematic review provides a valuable synthesis of the current knowledge on the biology, ecology, and management of poisonous *Pimelea* species with a focus on the Australian livestock industry while identifying potential avenues for future research.

## 1. Introduction

Poisonous plant species are a major problem in the agriculture industry. Several Australian native plant species are poisonous belonging to major families including the rice flowers (Thymelaeaceae), the legumes (Mimosaceae, Fabaceae), nightshades and tobaccos (Solanaceae), cycads (sago palms) (Zamiaceae, Cycadaceae), the spurges (Euphorbiaceae), the saltbushes (Chenopodiaceae), the grasses (Poaceae), and the heliotropes (Boraginaceae). *Pimelea* poisoning is a well-known problem for the Australian livestock industry [1]. The genus *Pimelea* (commonly known as the rice flowers; Family Thymelaeaceae) is comprised of 140 species, of which 35 species are endemic in New Zealand with the remainder in Australia [2]. Of those endemic to Australia, there are four poisonous species and subspecies (*viz. Pimelea simplex* subsp*. simplex* F.Muell., *P. simplex* subsp. *continua* J.M.Black, *P. trichostachya* Lindl., and *P. elongata* Threlfall.) which all contain the diterpenoid orthoester toxin called simplexin [3]. This toxin is present in all plant parts with the highest concentrations often detected in the single-seeded diaspore, but this depends on the species. Green flowering plants contain the highest level of this toxin, but field weathering trials have also shown that dried plant parts still contain significant amounts of the toxin [4]. The concentration of simplexin has been found to vary significantly, not only among different species of the genus but also across various plant parts [4]. This variability highlights the importance of considering the specific *Pimelea* species and plant parts when assessing the potential risk of poisoning for livestock or other animals.

Poisonous plants pose a significant economic burden on the Australian livestock industry, causing health-related problems and leading to substantial economic impacts. In Queensland (QLD) alone, the cost of cattle deaths from poisonous plants amounted to AUD 10.2 million per year in the 1970s and 1980s [5], (*ca.* AUD 140 million in 2023 inflation adjusted). The impact of *Pimelea* poisoning is especially severe in western QLD and northern New South Wales (NSW), where land values and equity, as well as productivity, are at risk. In 2010 for instance, it was reported that in severe years impacts of *Pimelea* poisoning cost the Australian livestock industry around AUD 50 million annually due to production losses, additional cattle management, and controlling *Pimelea* growth, among other factors [3]. The economic losses associated with *Pimelea* poisoning can include reduced livestock numbers and lower weight gain, leading to decreased profitability for farmers and an increased cost associated with supplementary feeding and veterinary care. According to a recent survey conducted in 2021 involving 32 affected producers, the average annual financial loss resulting from *Pimelea* poisoning has increased to AUD 67,000 [6]. A 2017 survey suggested that affected landholders must remove livestock from on average 3500 ha per property for six months due to *Pimelea* poisoning, representing a significant reduction in grazing land [7]. When *Pimelea* is abundant, it can significantly reduce the carrying capacity of a property as large areas of land become unsuitable for grazing due to the high risk of poisoning. In years such as 2006 and 2015–2017, where dry summers were followed by winter rain, the high abundance of *Pimelea* during the winter and spring likely had a severe impact on livestock productivity and the carrying capacity of grazing land [7]. The higher financial losses may also affect the livelihoods of livestock producers, potentially leading to a decrease in production and economic stability in the industry. The findings of this survey emphasize the importance of ongoing research and development of effective management strategies to reduce the impact of *Pimelea*-related diseases on livestock and the wider agricultural sector. In addition to the direct economic losses, the impact of *Pimelea* poisoning can also have long-term effects on the health and productivity of grazing land, further reducing the sustainability and profitability of farming practices in affected areas [6].

*Pimelea* poisoning outbreaks are challenging to predict and manage as they occur sporadically. Sparse vegetation in perennial pastures and intense grazing practices can trigger these events [8,9]. Currently, there are limited sustainable and effective methods to combat *Pimelea* poisoning, including restricting livestock access to *Pimelea*-infested paddocks, providing supplementary food, and very limited use of herbicides to control *Pimelea* plants under certain circumstances [10]. Therefore, further research is required to develop strategies to address the impact of these poisonous plants on the livestock industry. It is crucial to prioritize livestock health and the sustainability of the industry by exploring alternative approaches to pasture management and prevention of *Pimelea* emergence.

This review article provides a comprehensive overview of the biology, ecology, and management of poisonous *Pimelea* species, with a special focus on their impact on the livestock industry. This information will be useful for developing integrated management strategies that minimize the risk of livestock poisoning due to *Pimelea* while also promoting the ecological sustainability of *Pimelea* species. This review will serve as a valuable resource for researchers, land managers, and other stakeholders interested in the management of poisonous *Pimelea* species in the context of the livestock industry.

## 2. Distribution

**Figure 1:**
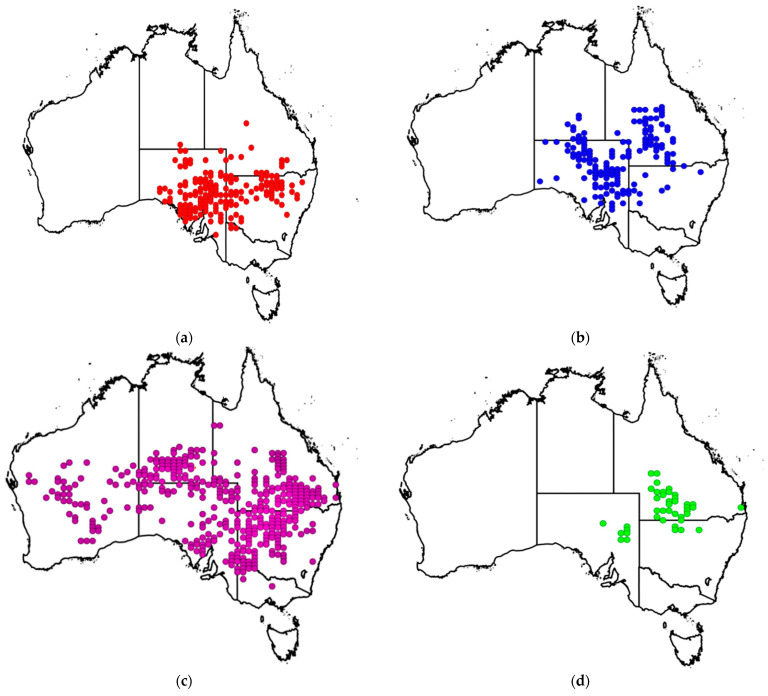
Distribution of the four poisonous *Pimelea* species across different States and Territories of Australia: (**a**) *P. simplex* subsp*. simplex* (**b**) *P. simplex* subsp*. continua* (**c**) *P. trichostachya* and (**d**) *P. elongata* in Australia. The data for maps were obtained from the Australasian Virtual Herbarium [13].

The four poisonous species and subspecies of *Pimelea* (*viz. P. simplex* subsp*. simplex*, *P. simplex* subsp. *continua, P. trichostachya*, and *P. elongata*) are all Australian native species, not found elsewhere in the world. These species have been reported to cause St. George or Marree disease [11,12]. *Pimelea simplex* has been found in NSW, QLD, Victoria (VIC), South Australia (SA) and the Northern Territory (NT). The two subspecies of *P. simplex* (Figure 1a,b) are distributed differently with some places where they co-exist. 

**Figure 2:**
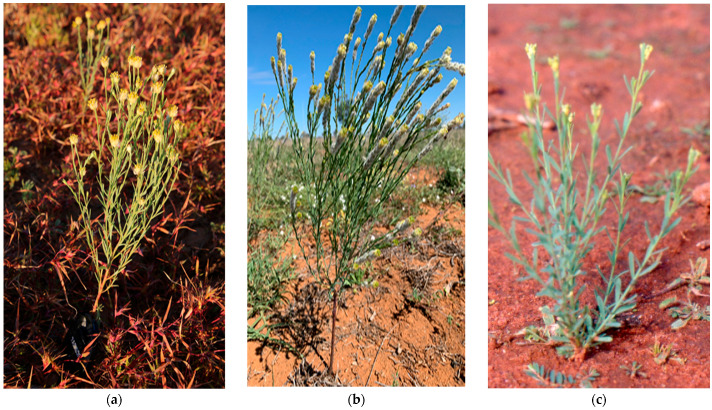
*Pimelea* plant species in field situations: (**a**) *P. simplex* subsp*. simplex* (**b**) *P. trichostachya* and (**c**) *P. elongata* [Source: Jenny Milson (**a**,**b**) and Richard Silcock (**c**) with permission].

According to herbarium records and several regional botanical publications, *P. trichostachya* is present in NSW [14], QLD [15,16], SA [17], Western Australia (WA) and in the NT [13]. (Figure 1c). However, there are no authenticated reports of poisoning associated with this species in WA and only a single report from the NT [18]. *Pimelea elongata* has a more restricted distribution in the regions of south-western QLD, north-western NSW and north-eastern SA (Figure 1d and Figure 2c). It is often abundant in the Mulga bioregion of south-western QLD where it can be found almost anywhere water ponds, including clayey ephemeral lakebeds [2].

## 3. Biology and Ecology

### 3.1. Common Names

*Pimelea trichostachya* is known as flax weed, while *P. simplex* subsp. *simplex* is commonly known as desert rice-flower and *P. simplex* subsp. *continua* as gibber rice-flower. *Pimelea elongata* is known as lakebed *Pimelea* [4].

### 3.2. Botanical Features

The botanical features of the four poisonous species and subspecies have been previously described by Bean [2]. A comparative summary of key botanical features is provided in Table 1. These features can be used for identification purposes, and they are helpful for ecological studies. The knowledge of the key botanical features also provides a basis for developing sound management strategies.

### 3.3. Preferred Habitat

The two *P. simplex* subspecies both grow on a range of soil types spanning from red desert loams to alkaline and heavy clays. In contrast, *P. elongata* is found on slightly acid to neutral soils of varying texture but mostly in ephemeral swamp areas, on lake edges, in places with impeded drainage or areas with perched water tables [4]. *Pimelea trichostachya* prefers to grow on acidic, red earth sandy loams or hard-setting grey clays in arid to sub-humid climatic conditions with substantial annual rainfall. 

There are two biotypes of *P. trichostachya*; a yellow-leafed form which is found more on the red earth sandy loams and a blue-leafed form found on the grey clay soils. *Pimelea trichostachya* is often found in buffel grass [*Pennisetum ciliare* (L.) Link] paddocks while *P. simplex* can grow well in Mitchell grass (*Astrebla* spp.) paddocks. These species do not tolerate shade and are rarely found where trees and shrubs are growing.

### 3.4. Environmental Requirements

All four poisonous species and subspecies are regarded as annuals [15,19]. They flower in spring and grow well in winter [20], with dry summers usually suppressing their establishment [21]. Their abundance depends on the prevailing environmental conditions and the intensity of inter- and intra-specific competition. 

Early summer rain may trigger re-shooting of mature *P. simplex* plants, but they are unable to withstand hot summer conditions. Nevertheless, *Pimelea* species have a hard diaspore coat, which allows them to tolerate very adverse conditions and remain viable in the soil for years [4].

Heavy shading restricts flowering of all four poisonous species, but plants have been found growing under partial light conditions. A minor rainfall event (20 to 30 mm) encourages *Pimelea* growth in a dry season. This can be found in gilgais (small, ephemeral lakes), watercourses, roadside drains and drainage courses across flat lands. Significant features of the climate in areas where the *Pimelea* species are found include winter rain and frosts, high rainfall variability (leading to the possibility of long periods between suitable rainfall conditions for diaspore germination and plant growth) and temperature extremes (long, hot summers and cold winter nights). Mature diaspores shed from the plant in early summer do not germinate at the first rains because of a hard diaspore coat, possibly imposing a physical dormancy [22]. In general, *Pimelea* species germinate during autumn, flower in the spring and mature diaspores are dropped directly on to the ground during early summer leading to localised high density *Pimelea* stands. There is ample circumstantial evidence to suggest that the density of *Pimelea* tends to be greatest where the pasture condition is poorest [14].

### 3.5. Floral and Reproductive Biology

**Figure 3:**
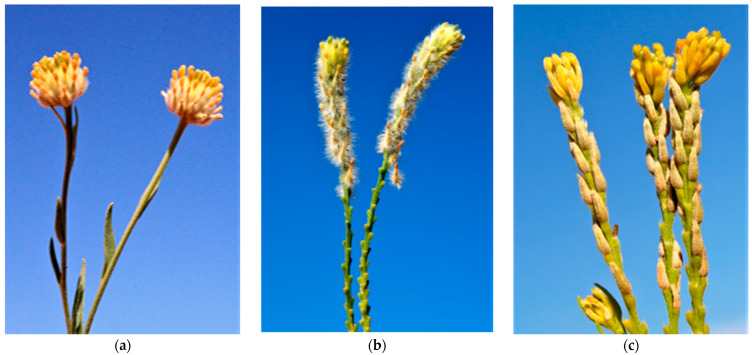
Flower heads of (**a**) *P. simplex* subsp*. simplex* (**b**) *P. trichostachya* and (**c**) *P. elongata* (Source: Jenny Milson with permission).

*Pimelea trichostachya* and *P. elongata* flower at any time of the year, irrespective of day length [19]; however, *P. simplex* flowers from late winter to late spring [2]. All four species produce many flowers. Pollination starts from the base and continues towards the tip of the flower head (Figure 3a–c). After fertilization has occurred, the ovule, now forming the inner fruit wall or endocarp, turns a deep blackish purple, which is occurring inside the floral tube and forming the outer fruit layers, the exocarp and mesocarp. During embryo enlargement, the embryo sac expands within the semi hardened endocarp. 

The embryo sac usually inflates without the embryo maturing within the now toughening endocarp. From each flower a single seeded fruit is formed (referred to as a diaspore). The diaspores of each *Pimelea* species have a distinctive, little extended knob on the tip, which is a deep yellow in colour, and can break off as the diaspore ripens [6]. After attaining physiological maturity, the diaspores are shed quickly through the formation of an abscission layer at their base. Diaspore numbers depend on the stage of plant maturity. According to Dadswell [9], a *P. trichostachya* population with an average plant density of six plants m^−2^ produced 1768 diaspores m^−2^ with each plant producing 288 diaspores [9]. Fresh diaspores are dormant and are hard to germinate even after scarification in laboratory conditions [22].

### 3.6. Diaspore Dispersal and Soil Seedbank

There are different modes for seed dispersal. Water is a major vector for seed dispersal; fresh diaspores are hydrophobic and float on water without imbibing water or when experiencing a certain level of abrasion, they will sink. Dirt within the water body can play a significant role in the abrasion process. After some time in water, some diaspores will still be floating while some will have sunk. Wind dispersal is also common as fresh diaspores are easily blown over the soil surface until trapped within soil cracks, or by litter, pebbles, and rocks [4]. Diaspores that fall on bare soil can become airborne in strong winds and travel over longer distances [4]. Light rainfall and dew can act as wetting agents to slow dispersal particularly in clay and silty soils. In these situations, diaspores often stick to the soil near to the parent plant and therefore, do not move long distances. Since dry diaspores are readily trapped by surface irregularities and plant litter *Pimelea* plants are often not found on scalded spots in paddocks [4].

Diaspores may also be spread through the faeces of moving flocks of sheep (*Ovis aries* L.) and goats (*Capra hircus* L.) since these livestock can consume *Pimelea* plants that carry viable diaspores [23]. Farm vehicles are also thought to be a mode of diaspore dispersal. The best strategy to prevent *Pimelea* dispersal is to control the plants before flowering and well before seed formation has occurred. Diaspores can remain dormant and viable in the soil seedbank for 2 years with the potential for longer-term persistence [24].

### 3.7. Seed Dormancy and Germination Ecology

The weathering effect on the diaspores of *P. simplex* subsp*. continua, P. trichostachya*, and *P. elongata* was investigated in a field study conducted across three States from 2007 to 2009 [4]. The study found that after 24 months of exposure to field conditions, the germination rates of *P. trichostachya* and *P. elongata* were higher as compared to diaspores that had been stored in a laboratory. This suggests that exposure to natural weathering conditions can improve the germinability of the diaspores of all three species. *Pimelea trichostachya* seed took more than 3 months to germinate presumably due to the presence of strong physical dormancy. *Pimelea simplex* diaspores presented an even deeper dormancy with no germination even after 9 months of weathering. Similarly, laboratory stored seeds of this species did not germinate for at least 18 months after storage [4]. *Pimelea elongata* took less than 2 months weathering to germinate as its diaspores presumably had a weaker physical dormancy. Diaspores are known to remain viable for long periods of time in the soil, withstanding extremely dry conditions, fire, land remodeling by bulldozing, cultivation or following land disturbance by animal hooves [22].

Given the significant dormancy mechanisms present in *Pimelea* diaspores, they often exhibit protracted germination behavior in the field. However, for laboratory work, this is not desirable, and pretreatments are usually applied to achieve uniform germination. Chemical stimulation approaches such as the use of gibberellic acid (GA_3_) and smoke water imbibition have provided variable germination success [22]. Similarly, physical dormancy breaking treatments such as scarification, seed coat removal, cold stratification and heat treatment have all provided some levels of germination success [22]. However, cutting the seed coat was extremely difficult and it could damage the embryo, so it was ruled out as an impractical method to produce seedlings for plant growth studies [22].

A recent study on the germination ecology of *P. trichostachya* suggests that it has a complex dormancy mechanism that involves multiple factors. The study identified a physical component of dormancy that can be partially overcome by diaspore scarification, a metabolic dormancy that can be overcome by the application of the plant growth regulator GA_3_, and a suspected third mechanism based on a water-soluble germination inhibitor [25]. These results suggest that *P. trichostachya* has a sophisticated system for regulating its germination in response to environmental cues and conditions, which can complicate management efforts. Freshly shed diaspores would germinate after a period of ageing (possibly promoted by high temperatures) in the soil to breakdown diaspore tissues (e.g., the hard endocarp). Once dormancy is broken, *Pimelea* diaspores can germinate and form seedlings under a range of environmental and soil conditions [25].

These species tend to prefer moderate temperatures, and the presence of light is important for germination. *Pimelea* diaspores also require contact with or proximity to the soil surface for successful germination, and soil moisture should not be limiting. Mildly acidic soil conditions are preferred, although *Pimelea* can also grow in more neutral or alkaline soils. Understanding the environmental and soil conditions that are most favorable for *Pimelea* germination and seedling formation can help to guide the development of effective management strategies that target these specific conditions [25].

These optimal germination conditions coincide with the late summer/autumn climatic period in most habitats of these species in Australia. Moisture plays a significant role in successful germination and establishment of most *Pimelea* species. *Pimelea* diaspores usually germinate in higher rainfall areas, or in dry seasons after rainfall of 20 to 30 mm where runoff water is ponded. Moderate rainfall during the late summer/autumn period often triggers the germination process. Hence, the recruitment and survival of *Pimelea* plants are generally higher in southern QLD, as compared to northern regions due to differences in climate. This means that the environmental conditions in the south are more conducive to *Pimelea* growth, survival and reproduction, resulting in higher population densities and more persistent infestations. The lower rainfall and higher temperatures in the north during the optimum window of *Pimelea* germination can limit their establishment and survival. Understanding these regional differences is crucial for developing effective management strategies that are tailored to the specific conditions of each area [9].

## 4. *Pimelea* Poisoning

*Pimelea* poisoning, commonly referred to as “*Pimelea* poisoning syndrome”, occurs when livestock particularly cattle consume *Pimelea* plants. Poisoning has been reported across the drier regions of QLD, western NSW, and northern SA [4]. Toxic *Pimelea* species are distasteful to livestock and are usually avoided but become poisonous when substantial amounts are ingested [20]. Dried stalks consumed with other pasture species may also lead to poisoning [26]. When signs of *Pimelea* poisoning appear, the current strategy is to isolate or take animals to non-infested areas [27].

### 4.1. Symptoms of Pimelea Poisoning in Cattle

A relationship between disease and the presence of certain *Pimelea* species was not recognized until the late 1960s. Clark [28] investigated the process by which the disease developed by introducing finely milled *Pimelea* plant material into the rumen of cattle by a stomach tube. After 3 days of treatments, the cattle that had received more than 50 mg of the dried material kg^−1^ of their body weight day^−1^, exhibited jugular distension, diarrhea and mucous discharge from the eyes and nostrils [28]. An oedema or swelling of the neck and brisket also occurred and then they died within 7 days. Lower doses (25 or 15 mg kg^−1^ of body weight day^−1^) took more time (from 2 to 28 weeks) to show recognizable symptoms and intoxication of the animals. The gross post-mortem and clinical findings in field cases vary considerably [29,30]. The affected animals showed their walk becoming lethargic, giving the impression of abdominal or thoracic pain. With progression of the disease, the oedema spreads to the base of the neck, brisket, and forelegs. Clark [28] also reported that intravenous injection of plant extracts led to increased pulmonary resistance, and deduced that the root cause of the observed pulmonary oedema and heart failure was due to prolonged constriction of pulmonary venules.

Three major pathways have been identified for *Pimelea* poisoning of cattle. These pathways include, (1) activation of the protein kinase C enzyme which affects myosin, and results in the contraction of the walls of the thick muscled pulmonary blood vessels with a resulting pressure buildup, with heart impacts and then fluid leakage causing the characteristic subcutaneous oedema associated with *Pimelea* poisoning; (2) increases in the volume of blood exacerbates the circulatory issues, diluting red blood cells, and as a result, the concentration of oxygen carried to tissues falls leading to anemia; (3) intestinal tract damage that results in diarrhea. It is thought that because sheep and goats have less developed pulmonary blood vessel walls, they are less affected by circulatory issues and only experience the diarrhea impacts [4].

### 4.2. Toxins Isolated from Pimelea Species

Simplexin, a daphnane-like diterpene has been isolated from several *Pimelea* species including *P. trichostachya*, *P. simplex* [26] and *P. prostrata* [3,31]. Simplexin was found in variable amounts, depending on plant part and growth stage [32]. Similarly, daphnane-like orthoesters have been isolated from various *Pimelea* species [33,34]. The concentration of simplexin has been reported to significantly vary among three poisonous species of *Pimelea* and across different plant parts (Table 2). Significantly higher concentrations of simplexin were reported in *P. elongata* and *P. trichostachya*, especially highest in their flowers and roots when compared with other plant parts [4]. In *P. simplex*, flower heads and roots had similar simplexin levels, while leaves had lower amounts, and branches and stems contained trace amounts.

### 4.3. Stock Susceptibility

In general, cattle are susceptible to *Pimelea* poisoning and develop the full range of clinical symptoms, irrespective of breed, sex, and age [4]. However, the animals aged 18 to 24 months were most commonly affected by poisoning. While cases of *Pimelea* poisoning are more commonly reported in adult cattle, it is possible for young calves as young as 3 months old to be affected as well [29]. Similarly, poisoning occurs in home-bred as well as introduced cattle, British breeds as well as Brahmans and crossbreds. Poisoning occurs less frequently in steers, bullocks, and calves than in cows, bulls, heifers and weaners [35]. Home bred stock usually avoid grazing due to the unpalatable nature of *Pimelea* plants, especially when they are green. This may explain why stock that are introduced into *Pimelea* infested areas are more likely to show symptoms of disease than home bred cattle.

Sheep can be affected by eating green *Pimelea* causing profuse diarrhea, which may be lethal; however, sheep and goats do not exhibit the oedema [4,28]. Horses (*Equus caballus*) are also susceptible to the *Pimelea* toxins, which can be fatal. Weaver [36] reported that horses exposed to *P. simplex* developed severe oedema on the head, neck and brisket and liver lesions (similar to those seen in cattle) in the Marree area in SA. Likewise, Wilson [37] reported that exposure of horses to *P. trichostachya* near Roma in QLD resulted in them exhibiting the full range of symptoms, including signs of circulatory failure, severe oedema, and liver engorgement.

Stress also plays a significant role in the sudden death of some cattle while experiencing *Pimelea* poisoning. Dadswell [9] reported that many cows showing signs of poisoning died while calving in QLD and they suggested that the stress of calving played a crucial role in their death.

### 4.4. Pimelea Poisoning Management

The impact of *Pimelea* poisoning can be minimized by reserving an area of pasture with little or no *Pimelea* so that stock can be shifted from infested areas as soon as signs of the syndrome appear. There are also some management strategies that can be used to mitigate *Pimelea* poisoning. Any affected animals should be moved to areas free of *Pimelea* (i.e., a hospital paddock) and managed in a way that minimizes further stress. High protein supplements are recommended to activate rumen metabolism of severely affected animals. D’Occhio [38] suggested that gastric stimulant powder may be helpful to treat possible rumen stasis. Such powders can be drenched, after mixing with honey, water, and molasses. Drugs to treat and to sedate and relax may also provide symptomatic relief. Collins and Scholz [27] proposed to use drugs to promote the shrinking of blood vessels while Cantello [39] suggested intestinal protectants, for example, merican powder containing kaolin should be given for 1 to 2 days to treat dehydration and severe diarrhea. Supplementation blocks such as sulphur or urea [9], are also reported to decrease the impact of poisoning.

Farmers reported that it is imperative to avoid stressing of the affected animals because this may result in sudden death. For example, severely affected animals need to be transported to the hospital paddock, as even the stress of walking can kill the animals. *Pimelea* poisoning is really a cause of concern for the Australian livestock industry as a proper treatment needs to be developed.

## 5. Management of *Pimelea* Plants

Managing *Pimelea* species can be particularly challenging for livestock producers in Australia, especially those who manage large grazing properties. The cost and logistics of chemical or physical management can be prohibitive for many producers, particularly if the infested area is extensive.

Chemical control options for *Pimelea* species can be effective particularly when treatments are implemented at the early stage of plant development [40]. However, there may be concerns about the environmental impact of chemical control methods, particularly if they are not targeted and may affect non-target plant species. Physical control methods, such as pulling or slashing *Pimelea* plants, can also be challenging on large properties. These methods can be labour-intensive and may only provide short-term control, as *Pimelea* plants can quickly regrow from root fragments or diaspores.

Given these challenges, cultural management approaches may be more suitable for managing *Pimelea* species on large grazing properties. For example, implementing strategic grazing management practices, such as land rest and rotation, can help to reduce the impact of *Pimelea* on livestock while also promoting the growth of desirable pasture species [6]. Seedbank management practices, such as minimizing soil disturbance and promoting the establishment of competitive plant species, can also help to reduce the germination and establishment of *Pimelea* seedlings [6].

**Figure 4:**
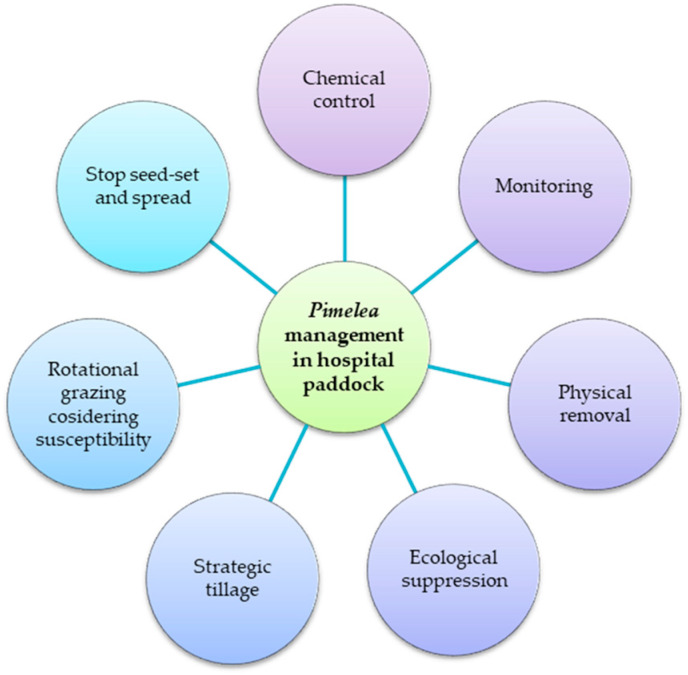
A multi-faceted approach to manage poisonous *Pimelea* species in hospital paddocks. The integration of suitable individual tactics could result in sustainable management.

Managing *Pimelea* species on large grazing properties requires a multi-faceted approach that takes into account the unique challenges of the landscape and the needs of livestock producers. Collaborative efforts between researchers, land managers, and industry stakeholders can help to identify the most effective management strategies for each specific situation. However, there are different methods to control abundance of *Pimelea* species in paddock dedicated for livestock quarantine (hospital paddocks) (Figure 4).

### 5.1. Preventive and Physical Control

There is no legislation to control or contain *Pimelea* plants in any jurisdiction as they are Australian native species. However, prevention and good farm biosecurity procedures could play a vital role in managing these poisonous plants. *Pimelea* fruits are wind and water dispersed and therefore, the physical hand pulling of plants must be done before flowering or before the diaspore is formed. Proper disposal of pulled plants is crucial to prevent them from re-growing or shedding diaspores. The most effective way to control *Pimelea* is to prevent its germination during the autumn and winter months. By avoiding the conditions that trigger *Pimelea* germination during this time, it is possible to effectively manage its growth and proliferation. The use of contaminated pasture seeds has been associated with the introduction of *Pimelea* in previously clean paddocks [4]. For instance, diaspores of *P. trichostachya* can mix with buffel grass seeds, resulting in ongoing infestations of this problematic species. Therefore, care must be taken while establishing new pastures to source clean, *Pimelea* free pasture seeds.

Different physical and mechanical control methods have been tried with variable success. For example, some producers have tried to suppress *Pimelea* plants through burning, however, anecdotal evidence suggests that this practice resulted in greater germination of *Pimelea* in the following season. Consequently, burning pastures is not recommended as it appears to encourage emergence of seedlings [4]. Slashing large plants generally does not lead to healthy regrowth; however, some regrowth may occur. When tips of small plants are damaged, they often develop new shoots from the internode and leaf junction. In addition, slashing leaves plant parts on the ground which still contain residual toxin and viable seed.

Shallow ploughing kills the plants but favours *Pimelea* recruitment from the soil seedbank in the long run. This is mainly because disturbances of the top-soil layers can encourage germination of dormant poisonous *Pimelea* diaspores [22]. Bare fallows have also been reported as a major source of *P. trichostachya* infestations in areas such as the Maranoa district in south-west QLD. Hence, the use of a fallow period is crucial, at the property or area-wide scale, to sustainably manage *Pimelea* species. Any physical or mechanical control option will have limited application due to the very large size of the livestock properties in the semi-arid and arid regions of Australia, making the process mostly cost prohibitive on a large scale.

### 5.2. Chemical Control

Various post-emergence herbicides are known to kill *Pimelea* in medic (*Medicago* spp.) or grass-based pastures, but such applications have only been studied in research trials [40]. Currently, there is only one minor use permit issued by the Australian Pesticides and Veterinary Medicines Authority (Permit No. 13549) [41] for the limited use of glyphosate and 2,4-dichlrophenoxyacetic acid (2,4-D) for the control of *Pimelea* in pastures. One of the most comprehensive studies on chemical control of *Pimelea* evaluated the efficacy of 13 post emergence herbicides (Table 3), selected based on their ability to control other similar herbaceous weeds [40].

Although glyphosate was able to kill *Pimelea* plants, it also killed desirable pasture species as well. Metsulfuron-methyl was the least expensive herbicide tested but less effective than 2,4-D. Metsulfuron-methyl, triclopyr + picloram and diflufenican all suppressed *Pimelea* growth, but in the case of *P. trichostachya* plants started to re-grow after some time. On the other hand, flumetsulam, aminopyralid + fluroxypyr, fluroxypyr, imazamox and MCPA + diflufenican were all found to be less effective in suppressing *Pimelea* growth and flowering.

To eradicate a *Pimelea* population from an area, repeated annual applications of herbicide would be needed, with a missed seeding event returning hundreds of diaspores m^−2^ to the soil [24]. Based on the findings of Silcock [40] further research on the post-emergence herbicides 2,4-D and metsulfuron-methyl is warranted given their efficacy and cost effectiveness in controlling *Pimelea*. To ensure that no poisonous plant residues are left behind, it’s important to determine the efficacy of various rates of herbicides on different growth stages of *Pimelea*, including seedlings, juveniles, and mature plants. This approach will enable the most effective herbicide and application rate for each growth stage to be chosen, while also minimizing the risk of environmental contamination.

Furthermore, there are possible complications with potentially increasing the chance of poisoning since the residual toxin remains in the dead plant material [32]. Thus, landholders are keen for investigations into the use of the pre-emergence herbicide tebuthiuron, as it is an herbicide that they are familiar with and use for controlling other vegetation types within *Pimelea* infested areas (Marie Vitelli, AgForce personal communication). A pot trial investigated the effect of tebuthiuron as a preventative treatment for *Pimelea* seedling emergence [6]. The study demonstrated that tebuthiuron was effective in preventing the emergence of *P. trichostachya* seedlings. Both a 10% granular and a 20% pelleted product of tebuthiuron were found to be effective, although the 10% granular tebuthiuron was marginally more effective at lower rates. Any seedlings that did manage to emerge eventually died within 7 days, and before they could leave any poisonous plant residues on the soil surface. The results of the pot trial are promising, but a field trial in a *P. trichostachya* infested paddock is necessary to validate the efficacy of tebuthiuron. It is suggested that testing of the commercially available tebuthiuron pellets should be sufficient, but the recently released liquid tebuthiuron product could also be considered to see if it provides improved efficacy.

### 5.3. Competitive Pastures

Pasture cropping had a significant suppression effect on weed density and diversity and, this can be used as a valid weed management tool [42]. Furthermore, sparse pastures usually encourage germination and seedling establishment due to low competition though there are reports that *P. trichostachya* can grow well in dense buffel grass pasture [4]. Plants growing in a community also suffer growth suppression by other plants. For example, observations of wild parsnip (*Trachymene* spp.) reported to suppress the growth of *P. elongata* under field conditions [4]. Scanty pastures usually encourage germination and seedling establishment of *Pimelea* due to low competition. For instance, Mitchell grass (*Astrebla lappacea* Lindl.) and wiregrass (*Aristida stricta* Michx) offer little competition during the early phase of their growth and development; however, competition starts in their later stages of growth [4].

The recent study by Saleem [6] suggests that Rhodes grass (*Chloris gayana* Kunth.), buffel grass, sabi grass (*Urochloa mosambicensis* Hack.), and Premier digit grass (Premier *digitaria eriantha* Steud.) were effective in suppressing the growth of *P. trichostachya*, and this may be a useful management tool in hospital paddocks. The results of the study indicated that, other than buffel grass, the tested grass species grew rapidly and suppressed the height, branch production, and biomass production of *P. trichostachya*, particularly in spring conditions when *P. trichostachya* usually grows well. Findings of this study suggested that incorporating these grass species in pasture systems could be a promising approach to help manage *Pimelea* species and to slow their spread, providing the prevailing environmental conditions are suitable for their establishment and growth. Further research is needed to determine the optimal conditions for using these grass species and to evaluate their effectiveness in different environments and management practices, and whether they are effective in controlling the spread of *Pimelea* species in other settings beyond hospital paddocks.

The suppressive ability of a pasture or crop species on *Pimelea* species is an important factor to consider in the management of these poisonous plants. Understanding the suppressive effects of different plant species on *Pimelea* spp. can help to inform the development of integrated management strategies. By using competitive pastures or crops, land managers can improve the productivity and sustainability of their grazing systems while simultaneously mitigating the risks associated with *Pimelea* poisoning.

### 5.4. Integrated Management

A producer survey conducted in 2021 found that landowners had the greatest success in managing *Pimelea* by adopting an integrated weed management (IWM) approach [6]. This approach involved planting strongly competitive pasture species, hand weeding in hospital paddocks, chemical control when *Pimelea* populations were small and grazing those affected paddocks with sheep or goats. Landowners reported that incorporating multiple control methods helped to reduce *Pimelea* infestations and maintain low levels of poisoning in their pastures. They also emphasized the importance of regular monitoring, to enable early detection, to effectively manage *Pimelea* infestations. Among the different methods used; rotation grazing was the most popular, followed by the application of herbicides. Some respondents summarised their approach as follows; generate greater ground cover as much as possible by growing trees, grass, and/or legumes, thus reducing the opportunity for *Pimelea* to germinate. Some landowners suggested burning the plants once established. One respondent suggested deep ripping to bury *Pimelea* diaspores. Others suggested that where properties had been subdivided into many paddocks, stock to be rotationally grazed. Meanwhile, many respondents had given up on trying to control *Pimelea* due to the large size of their properties and the areas of land that get infested. Some possible components of an IWM strategy for *Pimelea* species might include:

1. Monitoring: Regular monitoring of *Pimelea* populations can be highly beneficial in identifying the extent of their population size and any changes in their distribution over time. This approach can help to detect new infestations and track the success of control measures. By monitoring populations over time, it will provide insights into the ecology of *Pimelea* and the factors that influence its growth and spread, which can help inform more effective management strategies in the future. Additionally, early detection and rapid response can help prevent the establishment and spread of new infestations, which can save time and resources in the long term.
2. Cultural management: Strategies that modify the physical or biological conditions of the infestation site, such as altering the intensity of grazing can be highly effective in reducing the competitiveness of *Pimelea* populations. Planting trees to provide shade to inhibit germination can also be a pragmatic long-term strategy.
3. Chemical management: Herbicides can be effective in controlling *Pimelea* populations, but it’s essential to use them with care to avoid harming non-target species and the environment. It’s crucial to select an appropriate herbicide and application rate and to apply it during the appropriate growth stage of *Pimelea* to maximize its effectiveness.
4. Seedbank management: Adopting strategies that prevent the build-up of *Pimelea* seedbank, such as management before flowering, can be highly effective in reducing the persistence of this species over time. By detecting and removing plants before they can produce diaspores, will significantly limit the number of viable diaspores going to the soil and in the longer term prevent the establishment of further plants. This proactive approach can help to minimize the spread and costly control measures in the future.

The development of effective IWM strategies for *Pimelea* species will require a thorough understanding of their biology and ecology [25], as well as careful consideration of the potential impacts of management actions on the environment. Integration of chemicals with different cultural techniques or mechanical approaches is imperative to suppress and manage *Pimelea* species in a sustainable way [6].

## 6. Conclusions and Recommendations for Future Research and Development

The development of effective IWM strategies for *Pimelea* control is an important area of research and policy work. By implementing these strategies, livestock producers can reduce the impact of *Pimelea* poisoning on livestock and their grazing land, ultimately improving the sustainability and profitability of their operations. Here, are some further broad recommendations to help implement effective management of *Pimelea* species to reduce impact on livestock production:

1. Develop better awareness campaigns and promotion of containment guidelines to limit the spread of *Pimelea* species. By raising public awareness about the risks associated with *Pimelea* species and educating individuals on the importance of limiting their spread, we can encourage greater responsibility and proactive measures in containing these plants. This may include the development of quarantine areas, the requirement of permits for plant transport, and the establishment of monitoring programs to detect and respond to outbreaks. By promoting these guidelines, we can better regulate the movement of *Pimelea* and limit its potential impact on livestock and other animals.
2. Conduct further research and policy work to gain the registration of further herbicides for the specific control of *Pimelea* species in pasture systems. By developing and registering herbicides that can selectively target *Pimelea* plants, we can minimize the damage to other plants and reduce the risks of herbicide-resistant strains. Conducting further research on the herbicides that have already been approved for use in *Pimelea* control can also help to improve their efficacy and safety. This may include identifying optimal application methods and dosages for different plant growth stages and environmental conditions. Further research efforts and policy work can play a critical role in promoting the registration and use of *Pimelea*-specific herbicides. This may involve working with regulatory agencies to streamline the approval process for new herbicides or advocacy for research funding and collaboration with regulatory bodies.
3. Develop suitable IWM strategies that can be implemented under a wide range of conditions. Such strategies should involve the integration of various control options, including the use of herbicides, physical control methods, establishment of competitive pastures, and strategic grazing. By combining multiple control options, IWM can offer more effective and sustainable control of *Pimelea*, while also minimizing the risk of herbicide resistance. Establishing competitive pastures can also be effective in reducing the growth and spread of *Pimelea*, by promoting the growth of alternative plant species that compete for resources. This can be achieved through techniques such as pasture renovation, soil fertility management, and appropriate grazing management. Strategic grazing, particularly grazing with less effected species such as sheep and goats can also play a key role in IWM, by using livestock to target and reduce the density of *Pimelea* plants. By grazing at specific times and intensities, livestock can prevent the growth and spread of *Pimelea* while promoting the growth of alternative plant species.
4. Encourage collaboration between researchers, land managers, and other stakeholders in developing and implementing new and effective management strategies for *Pimelea* species. Ensure that these strategies are grounded in scientific evidence and are tailored to the specific needs of local communities and ecosystems. It is important to monitor the effectiveness of management strategies over time and make adjustments as needed based on new evidence or changing circumstances. This can help to ensure that management efforts remain effective and sustainable over the long term management of *Pimelea* species.

## Figures and Tables

**Table 1:** Botanical features and distinguishing characteristics of *Pimelea simplex* subsp. *simplex*, *P. simplex* subsp. *continua, P. trichostachya* and *P. elongata*. Source: [2,4].

	*Pimelea simplex* subsp. *simplex*	*Pimelea simplex* subsp. *continua*	*Pimelea trichostachya*	*Pimelea elongata*
Distinguishing features	Floral tube hairs 0.6 to 0.9 mm long; rachis length 3 to 6 mm	Floral tube hairs 1.4 to 2.8 mm long; rachis length 6 to 15 mm	Narrow leaves; long patent hairs on the floral tube; linear rachis and short anthers	Leaves without visible venation; sparsely hairy; the floral tube only 2.4 to 3.0 mm long at anthesis; anthers 0.3 to 0.6 mm long
Growth habit	Annual forb	Annual forb	Annual forb	Annual forb
Inflorescence	Terminal inflorescence; spicate; leafy bracts absent with 25 to 50 flowers produced (=number of persistent pedicels)	Terminal inflorescence with 35 to 100 flowers produced	Terminal inflorescence with 45 to 85 flowers produced; leafy bracts absent.	Terminal inflorescence; spicate; leafy bracts absent with 17 to 42 flowers produced; leafy bracts absent
Reproductive habit	Bisexual	Bisexual	Bisexual	Bisexual
Flowers	Creamy coloured with a yellow fringe	Yellow	Yellow, but obscured by white hairs; outer surface with two layers of hairs; a very dense layer of short patent hairs	Green-yellow to yellow, but obscured by white hairs
Phenology	Flowers and fruits are recorded from July to November	Flowers and fruits are recorded from June to January	Flowers and fruits may be found at any time of the year	Flowers and fruits may be found at any time of the year.
Stems	Young stems sparsely hairy, hairs slender, somewhat shiny and transparent, appressed to antrorse. Longest stem hairs 0.6 to 0.9 mm long	Longest stem hairs 0.8 to 1.1 mm long	Young stems sparsely hairy, longest hairs 0.8 to 1.0 mm long, slender, somewhat shiny and transparent, appressed to antrorse	Young stems very sparsely hairy, longest hairs 0.3 to 0.6 mm long, slender, somewhat shiny and transparent, appressed to antrorse
Leaves	Alternate attachment; lamina narrowly elliptic with no veins visible,	Alternate attachment, apex obtuse or acute	Alternate attachment, narrow leaves; lamina narrowly elliptic, apex obtuse or acute	Alternate attachment, leaves without visible venation and very sparsely hairy on the lower surface
Seed	Straight to slightly curved; fatter at the base; variable in size; often with the hairs aligned into three or four longitudinal ridges, seed ovoid, ca. 2.9 mm long, black, surface foveolate.	Curved (banana-shaped) and variable in size and with hairs of variable length, seed 2.8 to 3.1 mm long	Pear shape, seed ovoid; 2.4 to 2.5 mm long; black; surface foveolate	Pear shape, seed ovoid; 2.3 to 2.5 mm long; black; surface foveolate

**Table 2:** The distribution of the toxin, simplexin in various plant parts of representative flowering specimens of three poisonous *Pimelea* species. Source: [4].

	Simplexin Concentration (ppm)		
	*Pimelea trichostachya*	*Pimelea simplex* subsp*. simplex*	*Pimelea elongata*
Flowers/diaspores	709	253	341
Branches	70	trace	161
Main stem	48	trace	195
Leaves	49	22	244
Roots	66	281	409

**Table 3:** Herbicide options evaluated to control *Pimelea* spp. at Balonne and Roma in QLD, Australia. All treatments included a non-ionic surfactant (BS1000) at 0.25% *v*/*v*. Source: [40].

Trade Name	Active Ingredient	Rates Applied (g a.i. ha^−1^)	Herbicide Group
Amicide	2,4-D amine	500, 1000	4
Buticide	2,4-DB amine	320, 640	4
Tordon	2,4-D/picloram	450/900, 112.5/225	4
Hotshot	Aminopyralid/Fluroxypyr	5/10, 70/140	4
Brodal	Diflufenican	125, 250	12
Broadstrike	Flumetsulam	10, 20	2
Starane	Fluroxypyr	150, 300	4
Roundup	Glyphosate	1080, 2160	4
Raptor	Imazamox	17.5, 35	2
Kamba M	MCPA/dicamba	1020/2040, 240/480	4
Tigrex	MCPA/diflufenican	62.5/125, 6.25/12.5	12
Brushoff	Metsulfuron-methyl	6, 24	2
Grazon DS	Tricloppyr/picloram	300/600, 100/200	4

## Data Availability

Not applicable.
